# Editorial: What is known and what remains to be discovered about bacterial outer membrane vesicles, volume II

**DOI:** 10.3389/fmicb.2022.929696

**Published:** 2022-10-03

**Authors:** Alejandro Yañez, Rafael A. Garduño, Araceli Contreras-Rodríguez

**Affiliations:** ^1^Facultad de Ciencias, Universidad Austral de Chile (INCAR), Valdivia, Chile; ^2^Interdisciplinary Center for Aquaculture Research (INCAR), Concepción, Chile; ^3^Department of Microbiology and Immunology, Dalhousie University and the Canadian Food Inspection Agency, Halifax, NS, Canada; ^4^Departamento de Microbiología, Escuela Nacional de Ciencias Biológicas, Instituto Politécnico Nacional, Mexico City, Mexico

**Keywords:** outer membrane vesicles, OMVs biogenesis, Gram-negative bacteria, OMVs, bacterial vesicles

## Introduction

In 2019–2020, Frontiers in Microbiology published a collection of 13 research articles and five reviews under Volume I of the specialized Research Topic entitled “What is Known and What Remains to be Discovered about Bacterial Outer Membrane Vesicles” (the collection of Volume I articles can be accessed at <What Is known and What Remains To Be Discovered About Bacterial Outer Membrane Vesicles | Frontiers Research Topic (frontiersin.org)>) (References to the 18 publications of Volume I are also given in the references section and grouped under the subsection “Volume I”).

Given the success of Volume I of this Research Topic (reflected in a collective total of more than 100,000 online views) and the rapid scientific evolution of the subject area, it was decided in 2020 to launch Volume II. In this editorial, we, the topic editors of both volumes, present a bird's eye view of the seven original research articles and two reviews that compose the research collection published under Volume II in 2020–2021. The collection of Volume II articles can be accessed at <What is known and what remains to be discovered about bacterial outer membrane vesicles, Volume II|Frontiers Research Topic (frontiersin.org)>) (references to the nine publications of Volume II are also given in the references section and grouped under the subsection “Volume II”).

## Bacterial membrane vesicles at a glance

In the 1960s, some reports described the presence of “blebs” or “vesicles” released from the surface of the outer membrane of Gram-negative bacteria (Bayer and Anderson, 1965). It was then thought that outer membrane vesicles (OMVs) were fragments of lysed cells. However, over time, it was demonstrated that OMVs were actually formed at and released from live whole bacterial cells. Early electron microscopy observations showed the presence of numerous 50- to 300-nm spherical OMVs associated with whole bacterial cells, and their content was initially determined by SDS-PAGE (e.g., Gamazo and Moriyon, 1987). Since OMVs are formed from the outer membrane of Gram-negative bacteria, many components derive from this envelope and from the periplasm, including LPS, outer membrane proteins, and phospholipids. In early years of bacterial membrane vesicle research, no one thought that OMVs were relevant to the physiology and virulence of bacteria. However, historically, investigators have found toxins, hydrolases, and even nucleic acids contained in OMVs, which suggested potential roles of OMVs in pathogenesis and environmental fitness.

With the advent of more refined analytical tools (under the proteomics umbrella), it became possible to evaluate the complete protein composition of OMVs isolated from different bacteria, which in turn contributed to a better understanding of the complexity of these nanostructures (Langlete et al., 2019; Blackburn et al., 2021). One of the main discoveries made possible by the refinement afforded by proteomics is that the presence of proteins in vesicles is not randomly determined but instead proteins are targeted (directed), and their sorting is controlled by still ill-defined mechanisms.

All the new information has brought were new questions. What triggers the release of OMVs? How are proteins in OMVs selected? How does vesicle release occur without altering the integrity of the bacterial cell body? Why do OMVs transport virulence factors? Finally, what is the cell's purpose for releasing “custom-loaded” vesicles? The answers to these questions have been the focus of recent research efforts in different laboratories worldwide. Parts of this research effort constitute the core of the publications of Volume II of “What is Known and What remains to be Discovered about Bacterial Outer Membrane Vesicles”, as summarized in the five themes presented below.

## Biogenesis and stability of bacterial membrane vesicles

Vesicle formation requires the insertion of hydrophobic molecules into lipid bilayers that act as “wedges” and force the lipid bilayers to curve outward. The type and size of these wedge-type molecules, as well as the number of these molecules that get inserted, determine the size (diameter) and number of membrane vesicles formed. Therefore, a typical approach to determine the involvement of given molecules in the production (or origin/biogenesis) of vesicles is to simply observe changes in these physical properties (vesicle diameter and number of vesicles produced) in the presence or absence of such given molecules. Bacterial strains or mutants that produce an increased number of vesicles are called “hypervesiculating”, and several vesicle biogenesis factors have been identified by studying hypervesiculating deletion mutants, for instance lipoproteins, OmpA, LPS, and phospholipids (Nagakubo et al., 2020; Ávila-Calderón et al., 2021). In the case of *Lysobacter* sp. strain XL1, it has been now established that the bacteriolytic enzyme AlpB (which forms part of the OMVs' cargo) plays a role in vesicle biogenesis, since deletion of the *alpB* gene alters the quantity and quality of OMVs produced by this bacterium (Kudryakova et al.). It is important to note here that because AlpB is part of the OMV cargo, it also plays a role in mediating microbe-microbe interactions, as will be explained in the next section.

Besides biomolecules, environmental physical factors can affect the production/biogenesis of OMVs as well as their stability and function. It is thus important to establish how membrane vesicles are affected by (and how stable they are under) different environmental conditions. For instance, it is known that stress factors like pH, temperature, oxidative stress, and even simulated extraterrestrial Mars-like stressors in the international space station, i.e., Martian pressure, atmosphere, and UV illumination, affect the biogenesis and (or) stability of vesicles produced by different bacteria (Klimentova et al., 2019; Podolich et al., 2020; Sarra et al., 2020). Shishpal et al. have demonstrated that in *Gardnerella vaginalis*, extracellular changes in pH result in (i) morphological changes in vesicles (shown by electron microscopy), (ii) altered protein composition of vesicles (showing the presence of protein chaperones), and (iii) loss of cytotoxicity of OMVs toward vaginal epithelial cells.

Finally, for *Shewanella vesiculosa*, a cold-adapted Antarctic bacterium that produces great quantities of vesicles, Baeza et al. performed high resolution flow cytometry, combined with cryo-electron microscopy, to demonstrate the production of several types of membrane vesicles including double-layered vesicles (known as inner-outer membrane vesicles) that contain DNA. These authors also found that the prophage-mediated explosive cell lysis in *S. vesiculosa* is key in mediating the biogenesis and release of single- and double-layered membrane vesicles, and proposed that the vesicles produced as a result of bacteriophage-mediated bacteriolysis have different properties than the vesicles produced by blebbing (Baeza et al.).

## Bacterial membrane vesicles as mediators of microbe-microbe and microbe-host interactions

Vesicles are involved in microbe-microbe and microbe-host interactions (reviewed by Caruana and Walper, 2020). Bacteria may interact with other microorganisms playing the role of competitors (Knoke et al., 2020). In such interactions, the production and release of antimicrobial factors (e.g., antibiotics, cytolysins, or toxins) are key. The packaging of these antimicrobial factors into OMVs can increase their effectiveness. That is, instead of secreting free antimicrobials into the extracellular milieu (which can easily diffuse, get diluted, or be degraded), bacteria can keep these factors contained/protected in vesicles, which can then fuse with membranes of other microorganisms to produce effects (van den Berg van Saparoea et al., 2020). One example of this type of interaction is that mediated by membrane vesicles produced by *Lysobacter* sp. This bacterium produces vesicles loaded with the bacteriolytic enzyme AlpB, which besides playing a role in vesicle biogenesis (see previous section), is also responsible for killing competing bacteria (Kudryakova et al.).

The internalization of bacterial membrane vesicles by mammalian host cells is a typical microbe-host interaction mediated by vesicles, as previously demonstrated with vesicles produced by the intestinal commensal *Bacteroides thetaiotamicron* and are internalized by gastrointestinal tract cells (Jones et al., 2020). Now, it has been shown that the unusually shaped tubular OMVs released by the intracellular bacterial pathogen *Francisella tularensis* are internalized by bone-marrow-derived macrophages by micropinocytosis, chlatrin-mediated endocytosis, or lipid raft-dependent endocytosis. These tubular vesicles contain virulence factors and bacterial immunomodulatory proteins but, upon internalization, show no obvious cytotoxity toward macrophages. Instead, the internalized tubular vesicles induced pro-inflammatory responses in macrophages and appeared to somehow mediate the entry of this pathogen into macrophages (Pavkova et al.).

## Role of bacterial membrane vesicles as immunogenic antigens

Through proteomics or by the use of specific antibodies, it has been determined that some bacterial protein antigens carried naturally into vesicles are able to elicit a protective immune response in mammalian hosts. Based on this finding, and the fact that the lipidic nature of membrane vesicles makes these nanostructures a natural delivery vehicle for antigens, membrane vesicles have been proposed as a platform for developing novel vaccines. Thus, the vesicles produced by a number of bacterial pathogens have been evaluated as acellular vaccines in animal models (e.g., Araiza-Villanueva et al., 2019; Aispuro et al., 2020; Ávila-Calderón et al., 2020). The flexibility/adaptability of OMV-based vaccines has now been demonstrated in vaccines against pertussis. Since the currently circulating clinical strains of *Bordetella pertussis* (the causal agent of pertussis or whooping cough) have an increased ability to form biofilms, Carriquiriborde et al. used OMVs produced by a clinical *B. pertussis* strain grown in biofilms to develop a 2nd-generation vaccine that not only is more effective than the vaccine based on OMVs produced by the planktonically-grown strain but also induces a tissue-resident memory immune response.

Although OMVs have advantages in the development of novel acellular vaccines, some methodological problems still need to be resolved, especially those related to vesicle yield during the purification process as well as reducing the presence of full-length immunotoxic LPS, which could induce an inflammatory reaction (and even shock) in the human host. Solutions for these imminent problems include the use of rough bacterial mutants (obtained by natural spontaneous mutations or by induced mutagenesis, which produces LPS lacking the O-antigen) (Araiza-Villanueva et al., 2019), and the development of novel production platforms to provide recombinant OMV-based vaccines using the generalized modules for membrane antigens (GMMA) approach, as explained in the thorough review of Mancini et al.

## The two opposing roles of bacterial membrane vesicles in relation to infection treatments

Clinical bacterial infections are typically treated by use of antibiotics. Therefore, on the one hand, effective delivery of antibiotics (or alternate natural or synthetic antimicrobials) to bacterial pathogens would aid in treatment of infections. On the other hand, the emergence of antibiotic resistance in bacterial pathogens is a deterrent in treatment of clinical bacterial infections. Interestingly, there have been reports implicating OMVs as effective delivery vehicles of antibiotics like the bacteriocin-loaded OMVs of *Lactobacillus acidophilus* (Dean et al., 2020), as well as reports implicating OMVs in mediating the antibiotic resistance of bacterial pathogens (e.g., Vitse and Devreese, 2020), thereby establishing the opposing roles that OMVs could play in relation to infection treatments.

Now, the subject of using OMVs as delivery vehicles of antibiotics (and other naturally produced antimicrobials) has been reviewed comprehensively, establishing OMVs as tools for treatment of bacterial infections (Collins and Brown).

On the opposite side of the spectrum of bacterial infection treatments, it has now been demonstrated that OMVs from *Salmonella enterica* sv. Typhi can transiently transfer polymyxin B resistance to susceptible bacteria in cocultures. This finding is relevant since polymyxin is used as a clinical treatment for infections when other antimicrobial treatments fail to eradicate multi-resistant strains (Marchant et al.). The mechanism proposed by Marchant et al. involves sequestration by OMVs of a soluble polymyxin from the extracellular milieu rather than transfer of antimicrobial resistance genes *via* DNA-loaded OMVs.

## The diversified cargo of OMVs includes DNA and RNA

As mentioned before, OMVs contain components of the outer membrane, as well as the periplasm. In this respect, proteins that have been secreted across the inner membrane into the periplasm of Gram-negative bacteria constitute a common OMV cargo. However, recently, it has been unequivocally shown that OMVs can carry DNA and(or) RNA (e.g., Ashrafian et al., 2019; Langlete et al., 2019; Ahmadi Badi et al., 2020). The demonstrated presence of DNA in membrane vesicles is indeed intriguing, and the current question researchers in this area are asking is how this large cytoplasmic molecule gets targeted, and transported into OMVs? Now, Aktar et al., using the non-mobilizable, high copy number plasmid pUC19 and hypervesiculating mutants of *Escherichia coli*, observed that defects in peptidoglycan synthesis (intrinsic by genetic defects or externally induced by addition of 1% glycine) are linked to the presence of increased copy numbers of pUC19 in membrane vesicles. These authors proposed that plasmid DNA reaches the bacterial membrane vesicles of peptidoglycan-defective *E. coli via* two possible routes (mechanisms). One route implies increased membrane permeation leading to leakage of cytoplasmic contents without lysis, and the other involves the formation of intermediate inner-outer membrane vesicles (Aktar et al.).

## Conclusion

The information presented in this Research Topic regarding bacterial membrane vesicles, provide but a sample of the abundant literature derived from recent research efforts in the field. The five themes that we used to group the nine articles published under Volume II of this Research Topic represent forefront areas of the field, where most of the research activity is currently happening. It is therefore our privilege to present the nine articles of Volume II, which are in combination with the 18 articles published in Volume I and clearly show that this Research Topic is becoming increasingly exciting, touching on many aspects of bacterial physiology and bacteria-eukaryote interactions.

## Author contributions

All authors listed have made substantial, direct, and intellectual contributions to the article and approved it for publication.

## Funding

AC-R was supported by fellowships from COFAA-IPN, SIP-EDI, and SNI-CONACYT. This study was supported by grant FONDAP INCAR No. 15110027.

## Conflict of interest

The authors declare that the research was conducted in the absence of any commercial or financial relationships that could be construed as a potential conflict of interest.

## Publisher's note

All claims expressed in this article are solely those of the authors and do not necessarily represent those of their affiliated organizations, or those of the publisher, the editors and the reviewers. Any product that may be evaluated in this article, or claim that may be made by its manufacturer, is not guaranteed or endorsed by the publisher.
